# New risk stratification for adjuvant nivolumab for high‐risk muscle‐invasive urothelial carcinoma

**DOI:** 10.1002/bco2.298

**Published:** 2023-10-27

**Authors:** Takafumi Saito, Kent Kanao, Kazuhiro Matsumoto, Keishiro Fukumoto, Daisuke Igarashi, Takayuki Takahashi, Go Kaneko, Suguru Shirotake, Koshiro Nishimoto, Ryuichi Mizuno, Masaru Ishida, Satoshi Hara, Mototsugu Oya, Masafumi Oyama

**Affiliations:** ^1^ Department of Uro‐Oncology Saitama Medical University International Medicine Center Saitama Japan; ^2^ Department of Urology Keio University School of Medicine Tokyo Japan; ^3^ Department of Urology Saiseikai Yokohamashi Tobu Hospital Tokyo Japan; ^4^ Department of Urology Kawasaki Municipal Hospital Kawasaki Japan

**Keywords:** adjuvant therapy, immune‐related adverse events, neoadjuvant therapy, nivolumab, urinary bladder neoplasms

## Abstract

**Objectives:**

We aim to evaluate the risk of recurrence after neoadjuvant chemotherapy followed by radical cystectomy, particularly in ypT2 disease in patients with urothelial carcinoma, because it is not clear if all eligible patients with high‐risk muscle‐invasive urothelial carcinoma should be treated with adjuvant nivolumab.

**Materials and Methods:**

We analysed the radiological and clinicopathological features, including cT and ypT stages, of 197 patients who had undergone two to four cycles of cisplatin‐based neoadjuvant chemotherapy and radical cystectomy without adjuvant chemotherapy. We stratified the risk of postoperative recurrence by these factors.

**Results:**

The median observation period was 29.6 (interquartile range, 11.4–71.7) months, and disease recurrence was observed in 58 patients. Multivariate analysis revealed that ypT stage (*P* = 0.019) and lymphovascular invasion (*P* = 0.015) were independent risk factors for postoperative recurrence. The ypT2 group (*n* = 38) had significantly better recurrence‐free survival than the ypT3 group (*n* = 41) (median recurrence‐free survival: not reached vs. 13.4 months, respectively, *P* = 0.005). In ypT2 disease, the cT2 and ypT2 group (*n* = 15), which was diagnosed as cT2 preoperatively and then diagnosed as ypT2 postoperatively, had significantly better recurrence‐free survival than the cT3/4 and ypT2 group (*n* = 23) (median recurrence‐free survival: not reached vs. 63.1 months, respectively, *P* = 0.034). There was no significant difference in recurrence‐free survival between the ypT ≤ 1 (*n* = 106) and the cT2 and ypT2 groups (median recurrence‐free survival: not reached in both, *P* = 0.962).

**Conclusion:**

Patients with cT2 and ypT2 stage have a relatively low risk of recurrence and thus have a lower need for adjuvant nivolumab, particularly those with ypT2.

## 1 INTRODUCTION

Cisplatin‐based neoadjuvant chemotherapy (NAC) followed by radical cystectomy (RC) is the standard treatment for patients with muscle‐invasive cN0M0 bladder cancer.^1^ NAC has achieved approximately 10%–35% reduction in death risk compared with RC alone.^2^, ^3^, ^4^, ^5^ Significantly more cases are down staged to ypT0 with NAC followed by RC than RC alone; however, most cases still have residual cancer.^2^, ^6^, ^7^ The Checkmate 274 trial revealed that adjuvant nivolumab significantly prolongs recurrence‐free survival (RFS) in patients with ypT2 to ypT4a or ypN+ who receive NAC.^8^ However, immune checkpoint inhibitors cause immune‐related adverse events (irAEs), with 17.9% of patients in the Checkmate 274 trial study experiencing grade ≥3 adverse events.

Several studies have revealed no significant differences in overall survival (OS) between the cT2 group with NAC followed by RC and RC alone.^2^, ^9^, ^10^ The 5‐year OS was also higher in both groups than in the cT ≥ 3. Furthermore, a recent study revealed that NAC did not have a significant survival effect on patients with cT2N0 who were downstaged to pT < 2.^9^ It is unclear whether adjuvant chemotherapy (AC) improves the prognosis of patients with cT2 who receive NAC and are likely to have a low risk of recurrence. Previous studies have investigated biomarkers for predicting responsiveness to immunotherapy.^4^, ^11^, ^12^ However, detecting these biomarkers is complicated and expensive, and difficult to use in daily practice.

In this study, using real‐world data, we analysed the prognosis stratified by cT stage and ypT stage, especially the prognosis of the cT2 and ypT2 stages. The Checkmate 274 trial could not analyse the prognosis stratified by cT stage and ypT stage, which could assist in predicting the effectiveness of adjuvant nivolumab therapy. Administering adjuvant nivolumab to patients with a low recurrence risk may be unnecessary due to irAEs.

## 2 MATERIAL AND METHODS

### 2.1 Patients

Medical records of patients who underwent RC between January 2007 and December 2021 at our institution and three affiliated institutions were retrospectively investigated after approval from the Institutional Review Board (Ethics Number 20140234), with other participating sites providing the necessary institutional data‐sharing agreements before initiation. We identified 215 patients who underwent more than two to four cycles of cisplatin‐based NAC and RC without AC and excluded those patients who were treated with non‐cisplatin‐based NAC. We excluded patients with concurrent or a history of upper tract urothelial carcinoma (*n* = 3) and a history of partial cystectomy (*n* = 1), those with missing preoperative data or incomplete follow‐up information (*n* = 10), or those who died perioperatively (*n* = 3), and a patient who had received neoadjuvant radiation therapy (*n* = 1). The final cohort included 197 patients.

### 2.2 Data collection

Clinicopathological data, including patients' age, sex, hydronephrosis, cT and cN stage, the number of NAC cycles, histologic type in RC specimens, ypT and ypN stage, lymphovascular invasion (LVI), and disease recurrence, were obtained from medical records. Clinical and pathological stages were determined according to the American Joint Committee on Cancer staging manual, 7th edition. The diagnosis of hydronephrosis was confirmed by ultrasonography, computed tomography, or magnetic resonance imaging.

### 2.3 Treatment and endpoints

Our study began at the time of RC. RC was performed for non‐metastatic muscle‐invasive bladder cancer, recurrent multifocal superficial disease refractory to repeat transurethral resection with intravesical therapy, or Bacille Calmette‐Gurein resistant carcinoma in situ. All patients received cisplatin‐based NAC and no patient received AC. Five patients underwent simultaneous unilateral radical nephroureterectomy and none developed ypT2 disease. Of the five, two patients experienced disease recurrence and developed ypT0 and ypT4 disease, respectively. One underwent simultaneous total pelvic exenteration. Postoperatively, patients were generally followed up every 3–4 months for 2 years, then every 6 months until 5 years, and annually thereafter. Follow‐up visits consisted of physical examination and routine serum and blood tests. Diagnostic imaging, including computed tomography of the chest, abdomen and pelvis with or without intravenous contrast, was performed every 6 months for 5 years and then annually or when clinically indicated. The endpoint of this study, which was the time of disease recurrence. Based on radiographic imaging, disease recurrence was defined as locoregional or distant metastasis. Secondary urothelial carcinoma was not regarded as a disease recurrence.

### 2.4 Statistical analysis

RFS curves were constructed using the Kaplan–Meier method, and compared using the log‐rank test. Univariate and multivariate analyses of clinicopathological predictors of disease recurrence were conducted using the Cox proportional hazards regression model. We examined how the clinicopathological factors influenced the disease recurrence, reported as hazard ratio (HR) and 95% confidence intervals (CIs). Patients were censored if they did not experience recurrence at the last follow‐up. A two‐tailed *P* < 0.05 was regarded as statistically significant for all analyses. All analyses were performed using SPSS Statistics for Windows, version 27.0 (IBM Corp., Armonk, NY, USA).

## 3 RESULTS

### 3.1 Clinicopathological characteristics

The participants median age (interquartile range) at RC was 70.1 (65.7–74.6) years, most were male (*n* = 154 [78%]), and median observation period was 29.6 (interquartile range, 11.4–71.7) months. The clinicopathological characteristics of the 197 patients are summarized in Table 1. Regarding the pathological stage, nearly half of the patients (*n* = 91) had muscle‐invasive disease and LVI was observed in 48 (24%) patients.

**TABLE 1:** Clinicopathological characteristics of the 197 patients.

Characteristics	
Age at RC, median (IQR)	70.1 (65.7–74.6)
Sex, *n* (%)	
Male	154 (78)
Female	43 (22)
Hydronephrosis, *n* (%)	
No	156 (79)
Yes	37 (19)
Unknown	4 (2.0)
cT stage, *n* (%)	
1	4 (2.0)
2	77 (39)
3	98 (50)
4	18 (9.1)
cN stage, *n* (%)	
0	163 (83)
≥1	33 (17)
Unknown	1 (0.5)
NAC cycles, *n* (%)	
2	78 (40)
3	81 (41)
4	38 (19)
Histopathology classification in RC specimens, *n* (%)	
Pure UC	125 (64)
Non pure UC	12 (6.1)
No residual tumour	60 (31)
ypT stage, *n* (%)	
0	60 (31)
1	46 (23)
2	38 (19)
3	41 (21)
4	12 (6.1)
ypN stage, *n* (%)	
0	170 (86)
≥1	22 (11)
Unknown	5 (2.5)
LVI, *n* (%)	
Negative	146 (74)
Positive	48 (24)
Unknown	3 (1.5)
Recurrence, *n* (%)	
No	139 (71)
Yes	58 (29)

Abbreviations: cN, clinical N; cT, clinical T; IQR, interquartile range; LVI, lymphovascular invasion; NAC, neoadjuvant chemotherapy; RC, radical cystectomy; UC, urothelial carcinoma; yp, yield pathologic..

### 3.2 Risk factors for postoperative recurrence

According to the univariate Cox regression analyses, cT stage, cN ≥ 1, non‐pure urothelial carcinoma in RC specimens, ypT stage, ypN ≥ 1 and LVI positive were significant predictors of postoperative recurrence (Table 2). The number of cycles was not a significant predictor of postoperative recurrence. Furthermore, according to the multivariate analyses using the backward stepwise selection method, ypT stage and LVI positive remained as significant predictors of postoperative recurrence. The HR for disease recurrence in the ypT stage group, with ypT ≤ 1 group as the reference, showed that the higher the stage, the higher the HR, with the lowest HR in the ypT2 group and the highest in the ypT4 group.

**TABLE 2:** Univariate and multivariate analyses for recurrence.

	Univariate analysis			Multivariate analysis		
Variables	HR	(95% CI)	*P*‐value	HR	(95% CI)	*P*‐value
Age at RC, per 10 years	0.96	(0.82–1.12)	0.592			
Sex						
Male	Reference					
Female	1.21	(0.67–2.17)	0.533			
Hydronephrosis						
No	Reference					
Yes	1.28	(0.69–2.37)	0.442			
cT stage						
2	Reference		0.001			
3	3.00	(1.56–5.76)				
4	4.17	(1.76–9.92)				
cN stage						
0	Reference					
≥1	2.36	(1.33–4.16)	0.003			
NAC cycles						
2	Reference		0.124			
3	0.71	(0.49–1.03)				
4	0.96	(0.68–1.37)				
Histopathology classification						
Pure UC	Reference					
Non‐pure UC	2.44	(1.10–5.42)	0.029			
ypT stage						
≤1	Reference		<0.001	Reference		0.019
2	2.75	(1.25–6.02)		1.78	(0.67–4.73)	
3	7.74	(3.90–15.4)		3.67	(1.40–9.60)	
4	12.8	(5.48–29.7)		4.93	(1.55–15.7)	
ypN stage						
0	Reference					
≥1	4.76	(2.57–8.84)	< 0.001			
LVI						
Negative	Reference			Reference		0.015
Positive	5.91	(3.49–10.0)	< 0.001	2.31	(1.17–4.54)	

Abbreviations: cN, clinical N; cT, clinical T; LVI, lymphovascular invasion; NAC, neoadjuvant chemotherapy; RC, radical cystectomy; UC, urothelial carcinoma; yp, yield pathological.

### 3.3 Recurrence‐free survival

**FIGURE 1:**
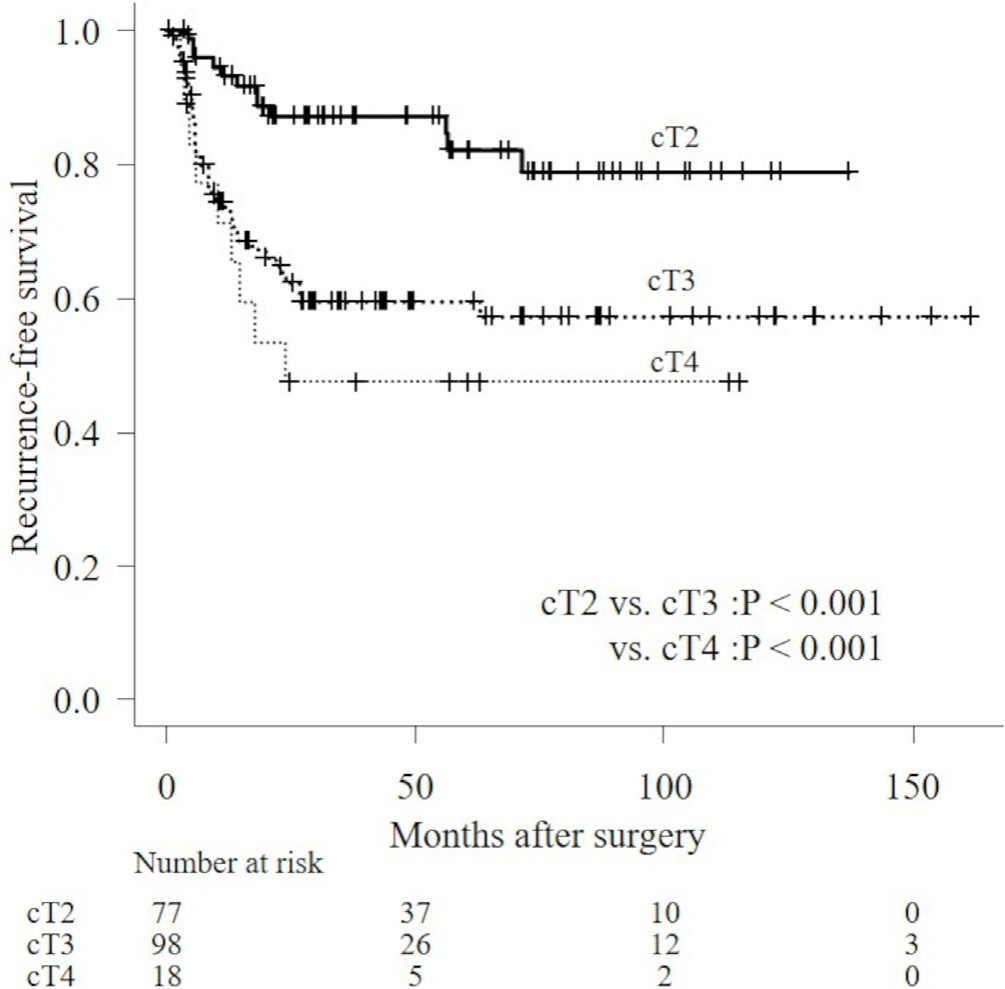
Kaplan–Meier analyses for RFS for patients with cT2, cT3 and cT4.

**FIGURE 2:**
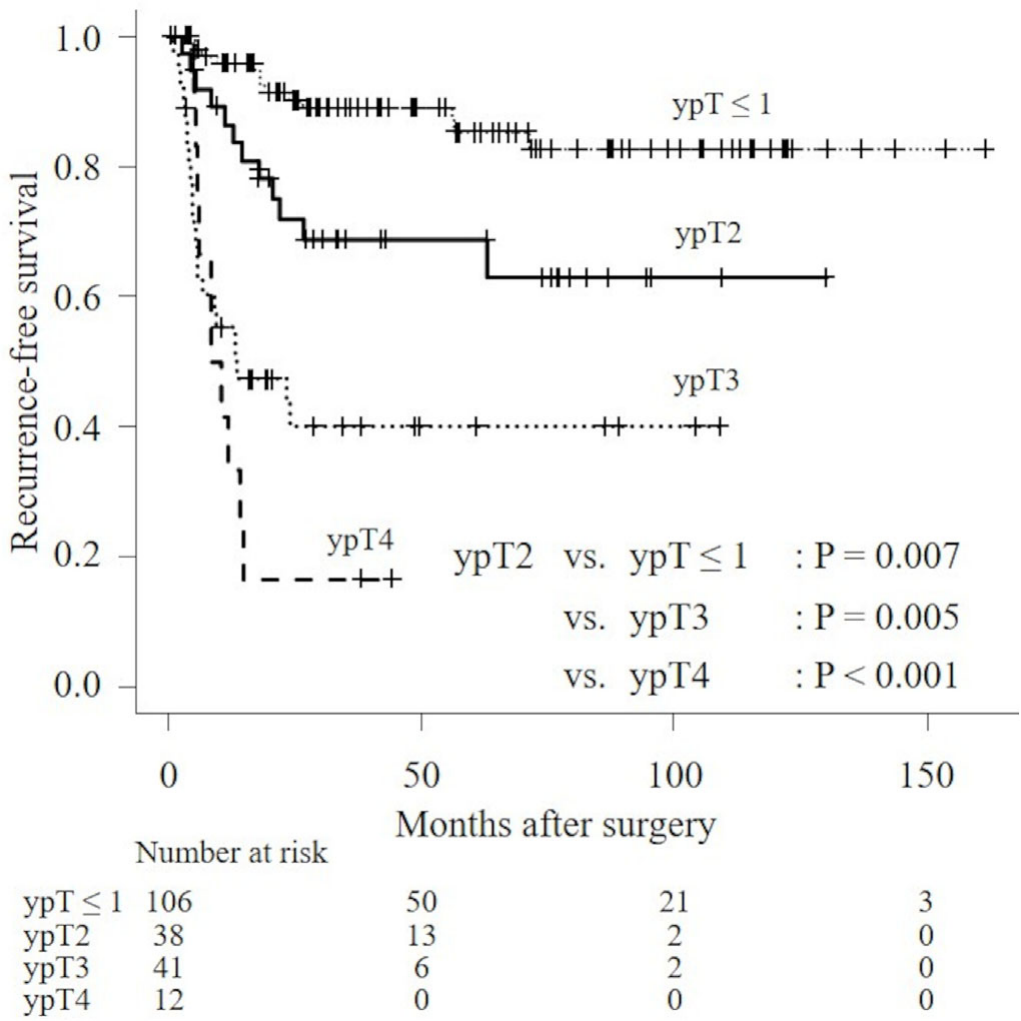
Kaplan–Meier analyses for RFS for patients with ypT ≤ 1, ypT2, ypT3 and ypT4.

Disease recurrence was observed in 58 patients (Table 1). Figure 1 illustrates the RFS at cT2 according to the log‐rank test; RFS at cT2 was significantly better than that at cT3 and cT4 (median RFS: not reached vs. not reached vs. 24.3 months, *P* < 0.001 and *P* < 0.001, respectively). Figure 2 illustrates that the RFS at ypT ≤ 1, ypT2, ypT3 and ypT4, according to the log‐rank test, are significantly different (median RFS: not reached vs. not reached vs. 13.4 months vs. 8.6 months, respectively). A higher disease recurrence was observed in the ypT3 and T4 groups than in ypT2 (*P* = 0.005, *P* < 0.001, respectively).

### 3.4 RFS subdividing ypT2 group with cT stage

**FIGURE 3:**
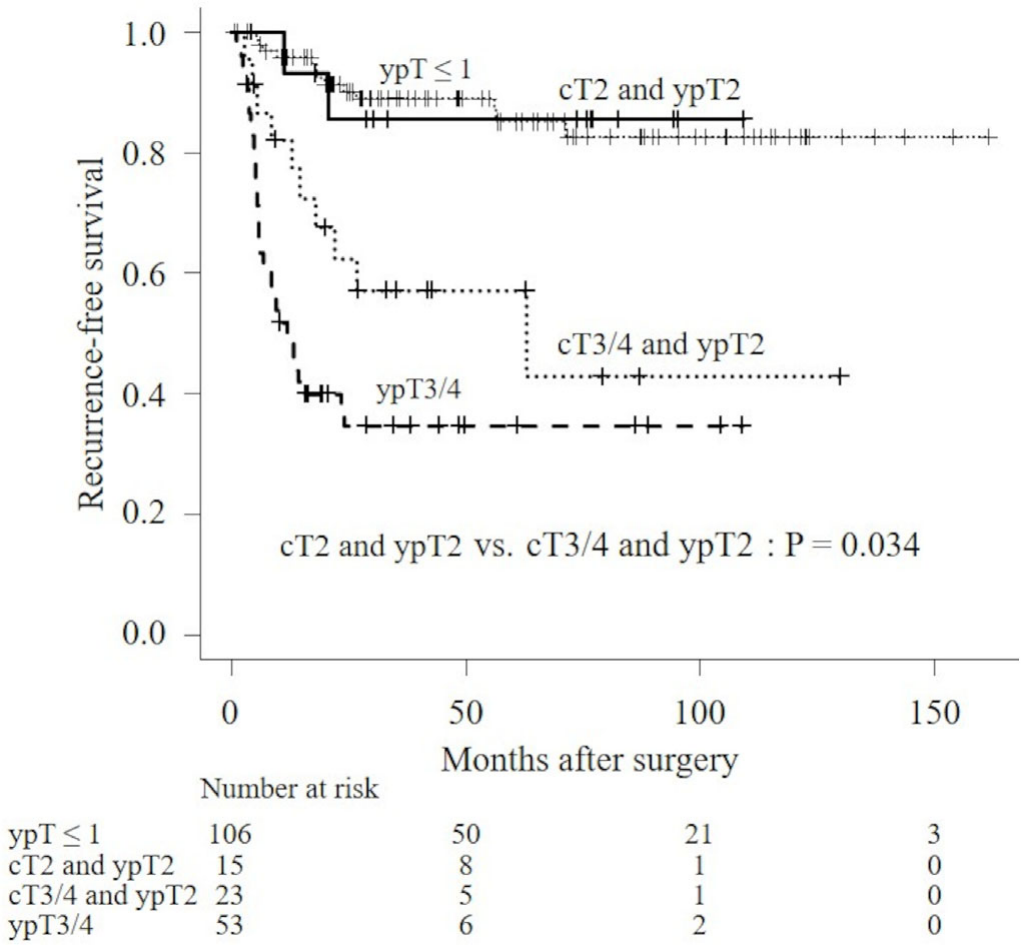
Kaplan–Meier analyses for RFS for patients with ypT ≤ 1, cT2 and ypT2, cT3/4 and ypT2 and ypT3/4.

**FIGURE 4:**
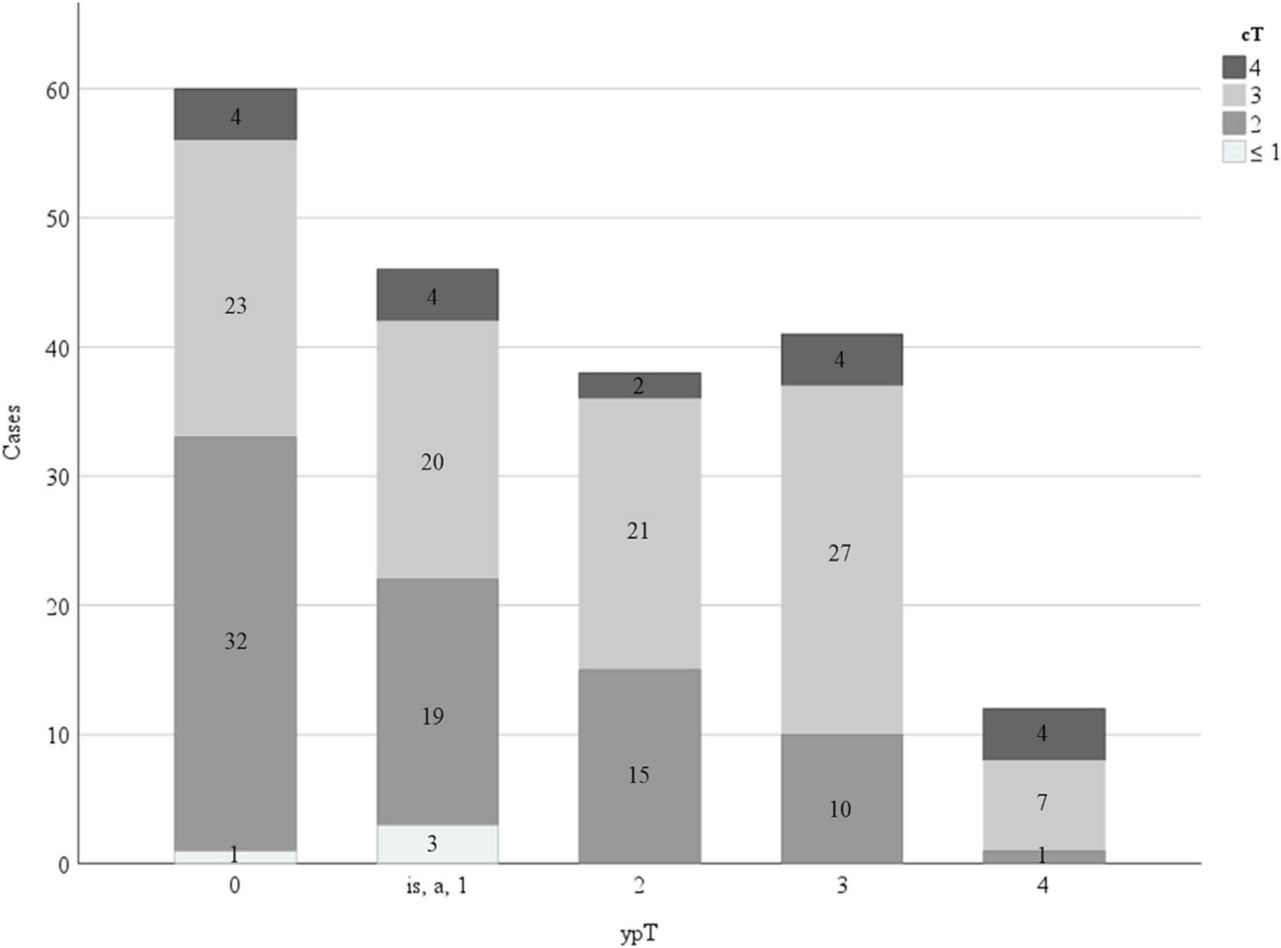
The relationship between cT and ypT stages.

Based on these results, we subdivided the ypT2 group into the cT2 and ypT2 group and the cT3/4 and ypT2 group. Of the 38 patients with ypT2 stage, cT2 disease was discovered in 15 patients, and cT3/4 disease was discovered in 23 patients. Figure 3 illustrates the RFS at ypT ≤ 1, cT2 and ypT2, cT3/4 and ypT2, and ypT3/4. There was no significant difference in RFS between the ypT ≤ 1 and the cT2 and ypT2 groups (median RFS: not reached in both, *P* = 0.962). Disease recurrence was observed in 13 and two patients in the ypT ≤ 1 and cT2 and ypT2 groups, respectively. There was a significant difference in RFS between the cT2 and ypT2 group and the cT3/4 and ypT2 group (median RFS: not reached vs. 63.1 months, *P* = 0.034). A higher disease recurrence was observed in 10 patients in the cT3/4 and ypT2 group than in the two patients in the cT2 and ypT2 group. There was a significant difference in RFS between the cT2 and ypT2 group and the ypT3/4 group (median RFS: not reached vs. 12.1 months, respectively; *P* = 0.001). Disease recurrence was observed in two and 33 patients in the cT2 and ypT2 group and the ypT3/4 group, respectively. Figure 4 presents the relationship between cT and ypT stages.

We also determined the RFS in the ypT2 group and compared it between the LVI negative and positive groups after excluding two patients with unknown LVI status from this analysis. Figure S1‐a illustrates the RFS for the LVI negative and positive groups among the patients with ypT2 disease. There was no significant difference in RFS between LVI negative and positive groups (median RFS: not reached vs. 26.9 months, *P* = 0.067). Moreover, we subdivided the patients in the cT2 and ypT2 group into LVI negative and positive groups to determine the RFS. We excluded a patient with unknown LVI status from this analysis. That patient did not experience disease recurrence. Figure S1‐b illustrates the non‐significant difference in RFS between LVI negative and LVI positive groups in the patients with cT2 and ypT2 disease. No patient in the LVI positive group experienced disease recurrence.

We determined the RFS of patients who underwent 3 or 4 cycles with NAC (Figure S2). Disease recurrence was not observed in the cT2 and ypT2 group. Meanwhile, disease recurrence was observed 6 patients in cT3/4 and ypT2 group. There was no significant difference in RFS between cT2 and ypT2 group and cT3/4 and ypT2 group (median RFS: not reached vs. 63.1 months, *P* = 0.095).

### 3.5 Risk factors for postoperative recurrence post subdividing ypT stage with cT stage

We subdivided ypT stage with cT stage into ypT ≤ 1 (reference group), cT2 and ypT2, cT3/4 and ypT2, ypT3, and ypT4, and included them in the univariate analysis. The ypT stage subdivided by cT stage was a significant predictor of postoperative recurrence (*P* < 0.001). According to the multivariate analyses using the backward stepwise selection method, ypT stage subdivided by cT stage (*P* = 0.016) and LVI (*P* = 0.018) were significant predictors of postoperative recurrence (Table 3). However, in the ypT stage subdivided by cT stage categories, based on the HR and 95% CI, only ypT3 and ypT4 were significant predictors of postoperative recurrence (HR = 3.75 [95% CI, 1.44–9.80] and 5.45 [1.75–17.0], respectively) while cT2 and ypT2 as well as cT3/4 compared with ypT ≤ 1 (reference group) showed non‐significant effects.

**TABLE 3:** Multivariate analyses for recurrence.

Variables	Multivariate analysis		
	HR	(95% CI)	*P*‐value
ypT stage subdivided by cT stage			
ypT ≤ 1	Reference		0.016
cT2 and ypT2	0.78	(0.16–3.80)	
cT3/4 and ypT2	2.53	(0.91–7.09)	
ypT3	3.75	(1.44–9.80)	
ypT4	5.45	(1.75–17.0)	
LVI			
Negative	Reference		0.018
Positive	2.25	(1.15–4.41)	

Abbreviations: cT: clinical T; LVI, lymphovascular invasion; yp, yield pathological.

## 4 DISCUSSION

In this study, we discovered that the cT2 and ypT2 group had a significantly lower risk of disease recurrence in the ypT ≥ 2 groups, especially the ypT2 group. We also observed no significant difference in RFS between the cT2 and ypT2 group and the ypT ≤ 1 group. These results support our hypothesis that the cT2 and ypT2 group, which is indicated for adjuvant nivolumab administration, has a low risk of recurrence and does not necessarily require adjuvant nivolumab administration due to irAEs. To our knowledge, no study has stratified the ypT2 stage with the cT stage and focused on the difference in risk of recurrence.

A few studies have examined the prognostic impact of the cT stage before RC with NAC and pathological downstaging. Schultz et al. analysed 111 patients for survival following NAC and reported no association between pathologic downstaging after NAC and survival improvement in the cT2‐T3a group.^7^ However, that study included patients from heterogeneous backgrounds for treatment after NAC (RC, partial cystectomy, exploratory laparotomy only etc.). Hermans et al. examined the effect of neoadjuvant treatment on pathological downstaging (≤(y)pT1N0) and OS in 5157 patients, divided into those with cT2N0M0 and cT3‐4aN0M0.^6^ NAC was significantly associated with pathological downstaging in both cT2 and cT3‐4a groups. However, in the cT2 group, NAC was not significantly associated with superior OS compared with RC alone, and the 5‐year OS was approximately 55% in both treatment groups. Faiena et al. also studied the effect of NAC on OS in 20 128 patients with cT ≥ 2 diseases who underwent RC.^5^ They discovered that NAC was not associated with OS benefit in the cT2N0 group that was downstaged to pT < 2. They revealed that downstaging did not lead to an improved prognosis for the cT2 group, who were likely to have localized disease. However, these studies did not analyse the prognosis of patients in the cT2 group before NAC and in the ypT2 group after RC with NAC. Our study demonstrated that patients eligible for adjuvant nivolumab administration in the cT2 and ypT2 group, who were likely to have organ‐confined disease and did not downstage, had a low risk of recurrence, especially in the ypT2 group.

We hypothesized that patients with a low risk of recurrence and unfit for adjuvant nivolumab due to irAEs may not need adjuvant nivolumab. Few studies have examined the benefits of AC. Leow et al. conducted a meta‐analysis to evaluate the prognostic impact of AC on stage ≥ pT2 disease.^8^ AC was significantly associated with improved RFS and OS. This result contradicts our hypothesis. However, that study did not reveal the prognostic impact of AC at every pathological stage. Therefore, we need to analyse the impact of AC in pT2, an organ‐confined disease, and pT3/4, a non‐organ‐confined disease. Chen et al. studied the impact of AC on OS in 3066 patients who underwent RC for any pT, N0‐1, or M0 bladder cancer.^9^ In patients with pT2N0, AC did not reveal a significant difference in OS compared with RC alone and may not significantly impact the prognosis of the patients with localized disease. Thus, patients with cT2 and ypT2 tumours, who are likely to have organ‐confined disease, may not necessarily benefit from AC and do not necessarily require adjuvant nivolumab.

The Checkmate 274 trial revealed that adjuvant nivolumab significantly prolonged RFS in patients with high‐risk muscle‐invasive urothelial carcinoma who had undergone radical surgery.^4^ However, this trial could not reveal a significant difference in disease recurrence or death with adjuvant nivolumab in the ypT2 group. Immune checkpoint inhibitors are used for advanced bladder cancer; however, they have little or no effect in many patients.^13^, ^14^, ^15^ Moreover, 17.9% of the patients in the Checkmate 274 study experienced grade ≥ 3 irAEs. Therefore, it is essential to establish a predictive index for immune checkpoint therapy efficacy. The cT2 and ypT2 group had a lower risk of recurrence among patients with ypT ≥ 2. In the cT2 and ypT2 group, adjuvant nivolumab‐free treatment can be one of the treatment options for patients who are unfit for adjuvant nivolumab due to irAEs.

Our study has several limitations. One limitation of this study is that the extent of lymph node dissection varied from patient to patient. Patients without extended lymph node dissection may have harboured lymph node metastases at the time of RC. Moreover, we could not discuss the influence of LVI on the patients' prognosis. In this study, in ypT2 disease, there was no significant difference in RFS between LVI negative and positive groups. Therefore, in this study, we developed a risk model with ypT2 stratified by cT stage. We also could not investigate the impact of LVI on recurrence in the cT2 and ypT2 group due to the small number of cases. This study had a retrospective and non‐randomized design. Hence, this study has inherent limitations of any retrospective study, such as heterogeneous patient groups and variable follow‐up schedules according to each institution. We could not analyse whether the cT2 and ypT2 groups also had a low risk of death. In this study, many institutions terminated the follow‐up prior to the occurrence of death, resulting in a low number of reported deaths, with potentials of bias. Hence, in this study, we only analysed the RFS. In our next study, we hope to investigate the impact of adjuvant nivolumab on cancer‐specific survival and overall survival especially in patients with cT2 and ypT2. We included patients with two to four cycles of NAC and two cycles of NAC may be insufficient as chemotherapy. Patients commonly have either missed/held doses or fail to complete treatment cycles owing to intolerance, renal impairment, or haematological complications.^16^ Furthermore, especially for the cT2 group, which is likely localized disease, there is a tendency not to undergo the four cycles of treatment due to concerns about delays to surgery and potential side effects. Also in this study, the proportion of patients with cT2 disease who underwent three or four cycles of NAC was lower than the proportion of patients with cT3 or 4 disease who underwent three or four cycles of NAC (Table S1). The proportion of patients with three or four cycles of NAC at cT2, cT3, and cT4 were 48.1%, 70.4%, and 61%, respectively. Moreover, in the univariate Cox regression analyses, the number of NAC cycles was not a significant predictor of postoperative recurrence (*P* = 0.124). The analysis of only the patients with three or four cycles of NAC may deviate from real‐world clinical practice; thereby, introducing bias. In this study, we found that the RFS at the cT2 and ypT2 group was significantly better than the RFS at cT3/4 and ypT2 group among patients treated with two to four cycles of NAC. In our subsequent study, we hope to investigate differences in recurrence rates between each cycle of NAC and ensure that the stratification is more applicable to clinical practice. Despite these limitations, this is the first report to stratify the risk of recurrence post‐radical cystectomy and NAC by ypT2 stage and cT stage, and our results are important for helping to guide a more accurate use of adjuvant nivolumab that is tailored to each patient's risk of recurrence.

In the future, validation with an external dataset comprising many patients will be essential to verify the significant differences in RFS between the cT2 and ypT2 group. Thus, we can gain a deeper understanding of predicting the effectiveness of adjuvant nivolumab therapy.

In conclusion, the cT2 and ypT2 groups have a low risk of disease recurrence and do not necessarily need adjuvant nivolumab due to irAEs. Therefore, for patients with cT2 and ypT2 tumours who are unfit for adjuvant nivolumab considering irAEs, adjuvant nivolumab‐free treatment can be one of the treatment options.

## AUTHOR CONTRIBUTIONS

*Conception and design*: Takafumi Saito and Kent Kanao. *Acquisition of data*: Takafumi Saito, Kent Kanao, Kazuhiro Matsumoto and Keishiro Fukumoto. *Analysis and interpretation of data*: Takafumi Saito and Kent Kanao. *Writing the manuscript*: Takafumi Saito and Kent Kanao. *Reviewing the manuscript*: Kazuhiro Matsumoto, Keishiro Fukumoto, Daisuke Igarashi, Takayuki Takahashi, Go Kaneko, Suguru Shirotake, Koshiro Nishimoto, Ryuichi Mizuno and Masaru Ishida. *Study supervision*: Masaru Ishida and Satoshi Hara, Mototsugu Oya and Masafumi Oyama.

## CONFLICT OF INTEREST STATEMENT

All authors have no conflict of interest to disclose.

## Supporting information


**Figure S1‐a.** Kaplan–Meier analyses for RFS for patients with ypT2 disease by LVI negative and positive groups.

**Figure S1‐b.** Kaplan–Meier analyses for RFS for patients with cT2 and ypT2 disease by LVI negative and positive groups.

**Figure S2.** Kaplan–Meier analyses for RFS in patients who underwent three or four cycles of NAC with ypT ≤ 1, cT2 and ypT2, cT3/4 and ypT2 and ypT3/4.

**Table S1.** Stratified analysis of the: Stratified analysis of the: Stratified analysis of the: Stratified analysis of the: Stratified analysis of the: Stratified analysis of the: Stratified analysis of the: Stratified analysis of the: Stratified analysis of the: Stratified analysis of the: Stratified analysis of the: Stratified analysis of the relationship between the cT stage and NAC cycles.
