# Supplementary figures and images for: New risk stratification for adjuvant nivolumab for high‐risk muscle‐invasive urothelial carcinoma
Source: BJUI Compass. 2023 Oct 27;5(2):281–8. doi: 10.1002/bco2.298 (PMC10869665; doi:10.1002/bco2.298)

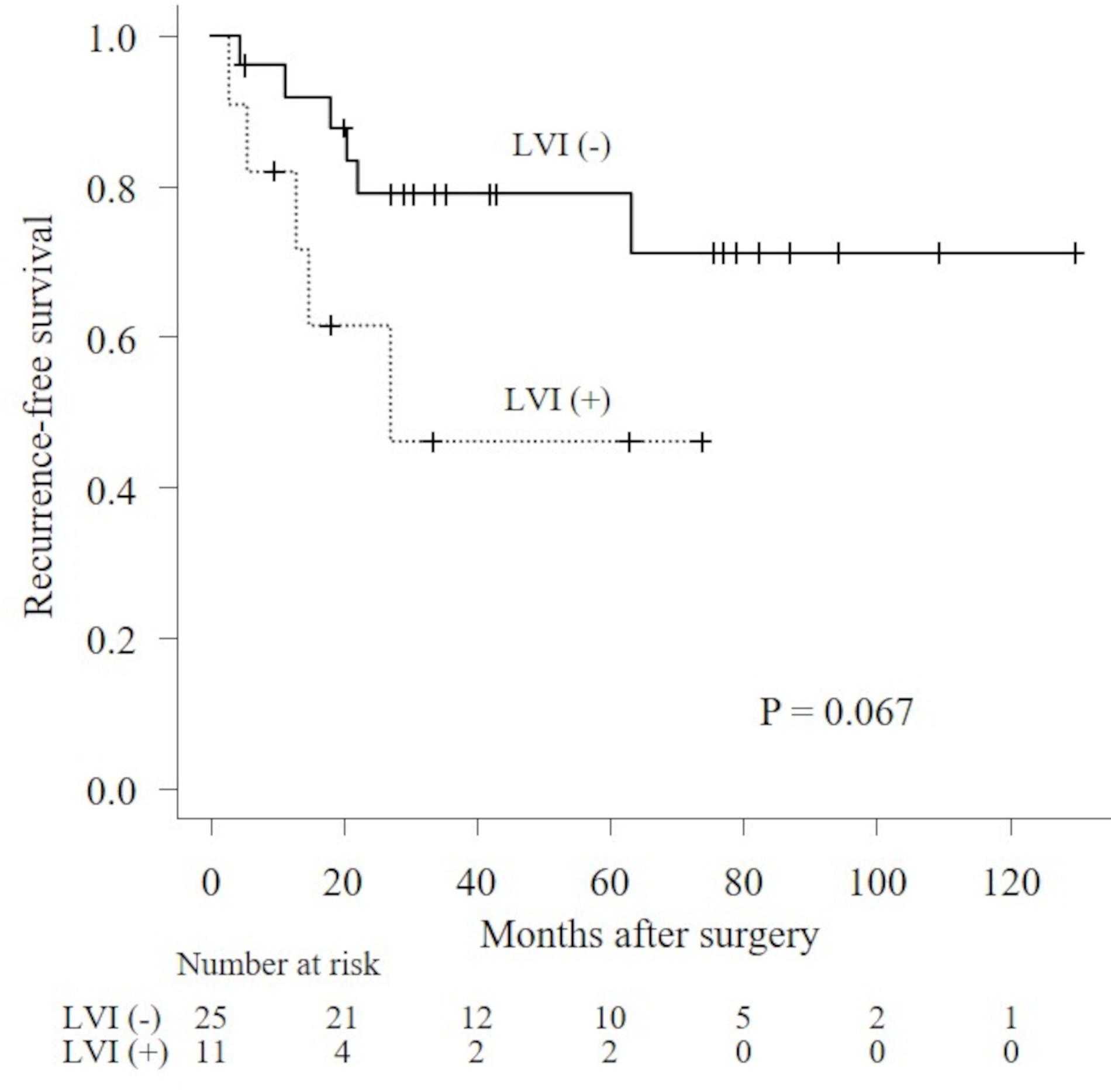

Supplement: Supplementary file 1 — Figure S1‐a. Kaplan–Meier analyses for RFS for patients with ypT2 disease by LVI negative and positive groups. [file BCO2-5-281-s003.jpg]

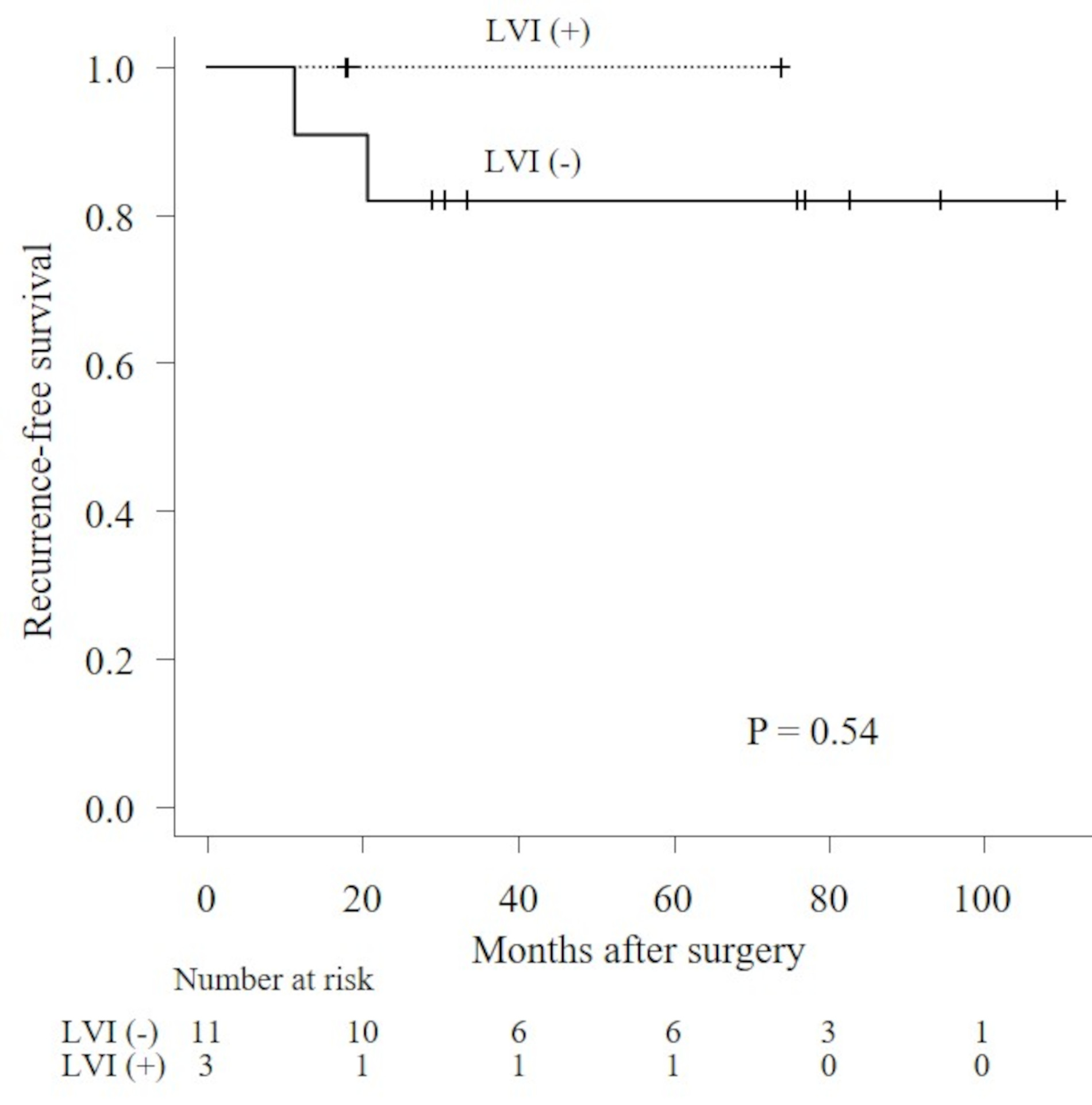

Supplement: Supplementary file 2 — Figure S1‐b. Kaplan–Meier analyses for RFS for patients with cT2 and ypT2 disease by LVI negative and positive groups. [file BCO2-5-281-s004.jpg]

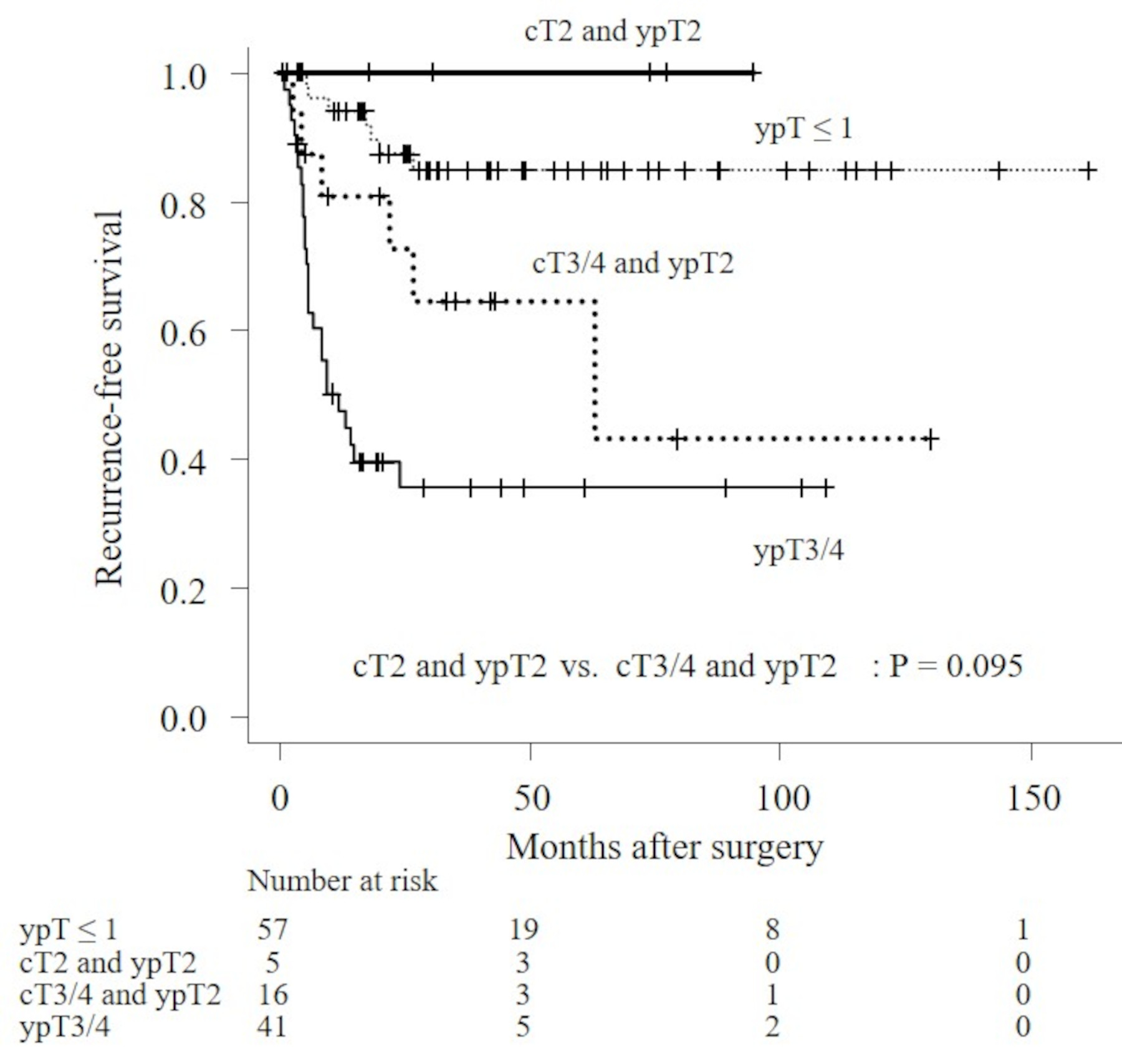

Supplement: Supplementary file 3 — Figure S2. Kaplan–Meier analyses for RFS in patients who underwent three or four cycles of NAC with ypT ≤ 1, cT2 and ypT2, cT3/4 and ypT2 and ypT3/4. [file BCO2-5-281-s002.jpg]
