# Supplementary material for: New risk stratification for adjuvant nivolumab for high‐risk muscle‐invasive urothelial carcinoma
Source: BJUI Compass. 2023 Oct 27;5(2):281–8. doi: 10.1002/bco2.298 (PMC10869665; doi:10.1002/bco2.298)
Supplement: Supplementary file 4 — Table S1. Stratified analysis of the: Stratified analysis of the: Stratified analysis of the: Stratified analysis of the: Stratified analysis of the: Stratified analysis of the: Stratified analysis of the: Stratified analysis of the: Stratified analysis of the: Stratified analysis of the: Stratified analysis of the: Stratified analysis of the relationship between the cT stage and NAC cycles. [file BCO2-5-281-s001.pdf]

Table S1 : Stratified analysis of the relationship between the cT stage and NAC cycles

cT stage	NAC cycles, n (%)			
	2	3	4	Total
cT≤1	2 (50)	2 (50)	0	4 (100)
cT2	40 (52)	25 (32)	12 (16)	77 (100)
cT3	29 (30)	47 (48)	22 (22)	98 (100)
cT4	7 (39)	7 (39)	4 (22)	18 (100)
Total	78	81	38	197

NAC, neoadjuvant chemotherapy; yp, yield pathological.
